# Protective effect of *Palmijihwanghwan* in a mouse model of cigarette smoke and lipopolysaccharide-induced chronic obstructive pulmonary disease

**DOI:** 10.1186/s12906-021-03453-5

**Published:** 2021-11-16

**Authors:** Eun Bok Baek, Jin-hyung Rho, Eunhye Jung, Chang-Seob Seo, Jin-Hee Kim, Hyo-Jung Kwun

**Affiliations:** 1 Department of Veterinary Pathology, College of Veterinary Medicine, Chungnam National University, 220 Gung-dong, Yuseong-gu, Daejeon, 34134 South Korea; 2 Herbal Medicine Research Division, Korea Institute of Oriental Medicine, Daejeon, 34054 South Korea

**Keywords:** Airway remodeling, Chronic obstructive pulmonary disease, *Palmijihwanghwan*, Cigarette smoke, Inflammation

## Abstract

**Background:**

*Palmijihwanghwan* (PJH) is a traditional medicine and eight constituents derived from PJH possess anti-inflammatory activities. However, the scientific evidence for its potential as a therapeutic agent for inflammatory lung disease has not yet been studied. In this study, we examined the protective effect of PJH in a mouse model of chronic obstructive pulmonary disease (COPD) induced by cigarette smoke (CS) with lipopolysaccharide (LPS).

**Methods:**

Mice received CS exposure for 8 weeks and intranasal instillation of LPS on weeks 1, 3, 5 and 7. PJH (100 and 200 mg/kg) was administrated daily 1 h before CS treatment for the last 4 weeks.

**Results:**

Compared with CS plus LPS-exposed mice, mice in the PJH-treated group showed significantly decreased inflammatory cells count and reduced inflammatory cytokines including interleukin-1 beta (IL-1β), IL-6 and tumor necrosis factor alpha (TNF-α) levels in broncho-alveolar lavage fluid (BALF) and lung tissue. PJH also suppressed the phosphorylation of nuclear factor kappa B (NF-κB) and extracellular signal-regulated kinase1/2 (ERK1/2) caused by CS plus LPS exposure. Furthermore, CS plus LPS induced increases in matrix metallopeptidase (MMP)-7, MMP-9, and transforming growth factor-β (TGF-β) expression and collagen deposition that were inhibited in PJH-treated mice.

**Conclusions:**

This study demonstrates that PJH prevents respiratory inflammation and airway remodeling caused by CS with LPS exposure suggesting potential therapy for the treatment of COPD.

**Supplementary Information:**

The online version contains supplementary material available at 10.1186/s12906-021-03453-5.

## Background

Chronic obstructive pulmonary disease (COPD) is a chronic inflammatory disease associated with persistent respiratory symptoms such as dyspnea, sputum overproduction, and cough [1]. COPD encompasses chronic obstructive bronchiolitis with subsequent fibrosis and closure of airways, and emphysema with expansion of airspaces [2]. COPD pathophysiology is characterized by persistent airway inflammation reflecting activated inflammatory cells (e.g., neutrophils, eosinophils, macrophages and lymphocytes) and pulmonary emphysema [3]. A history of exposure to inhaled toxic particles, smoke, such as cigarette smoke (CS), or gases is associated with the diagnosis of COPD [4]. Harmful particles of inhaled CS give rise to the airway inflammation and pulmonary damage that predominates in COPD patients [5]. CS is metabolized to numerous toxic chemical substances that generate reactive oxygen species (ROS), which play a major role in the pathogenesis of COPD. In fact, markers of oxidative stress, including nitrogen oxides and lipid peroxides, are elevated in the respiratory tract, blood, and lung tissues of COPD patients [6].

Current pharmacological therapy of COPD involves long-acting anti-muscarinic agents (LAMAs), long-acting β2 agonists (LABAs), inhaled corticosteroids, inhibitors of phosphodiesterase-4 (PDE4), such as roflumilast, and their combination therapies [7, 8]. Adverse drug reactions associated with several classes of drugs, including corticosteroids and PDE4 inhibitors, especially theophylline, have been reported, highlighting the need for alternative therapies, in particular, herbal products [9]. Herbal therapies for COPD are intended to mitigate symptoms, prevent recurrence, reduce the risk of comorbidities and improve the quality of life.

The levels of a number of specific inflammatory mediators, including several cytokines and chemokines that promote inflammatory process and influx immune cells into the lungs, are increased in lung tissues of COPD patients. These pro-inflammatory mediators are regulated by activation of nuclear factor-kappa B (NF-κB) and mitogen-activated protein kinases (MAPKs), particularly p38 MAPK and extracellular signal-regulated kinase (ERK)-1 and − 2 [1]. Increased ROS released from inflammatory cells, such as leukocytes and macrophages, are involved in the inflammatory progress in the lungs of COPD patients. They also generate chemokines the guide the inflow of excessive inflammatory cells, such as neutrophils, monocytes and lymphocytes, into the lung [10].

The herbal formula *Palmijihwanghwan* (PJH) is a traditional medicine in the Asian continent and also called Hachimi-Jio-Gan in Japan and Ba-Wei-Di-Huang-Wan in China [11]. It has been clinically used to treat many symptoms, such as diabetes, hypertension, back pain, edema, nephritis and dementia [12–15]. PJH is composed of eight herbal extracts including *Dioscorea batatas, Paeonia suffruticosa, Rehmannia glutinosa, Cornus officinalis, Aconitum carmichaelii*, *Cinnamomum cassia*, *Alisma orientale*, and *Poria cocos*. Among these herbal mixtures, for example, *Dioscorea batatas* shows anti-inflammatory effects and regulates energy metabolism, and 2, 7-dihydroxy-4, 6-dimethoxy phenanthrene isolated from *Dioscorea batatas* decreased the expression of proinflammatory cytokines and posed potential as a therapeutic agent for multiple inflammatory diseases, such as COPD [16, 17]. *Paeonia suffruticosa* improved lipopolysaccharide-induced acute lung injury in rats through anti-inflammation [18]. *Rehmannia glutinosa* is a major ingredient of PM014, which prevented inflammatory processes in mouse COPD models [19]. *Corni fructus,* which is a fruit of *Cornus officinalis,* showed therapeutic effects in ovalbumin-induced asthma animal model [20]. In addition, *Cornus officinalis* shows anti-inflammatory and analgesic effects [21]. *Aconitum carmichaelii* protected against sepsis-induced acute lung injury and has been used as an anti-inflammatory and analgesic therapies in Asian continent [22–24]. *Cinnamomum cassia* Blume is a popular traditional Chinese herbal medicine that has been used to manage respiratory tract disease and anti-inflammatory effect of *Cinnamomum cassia* was reported [25, 26]. *Alisma orientale* has been prescribed as a remedy for treating the diseases associated with body fluid dysfunction such as edema and inflammatory lung diseases [27]. *Poria cocos* exerted anticancer properties, especially against lung cancer [28].

Despite multiple previous reports on PJH constituents, none has investigated its protective effect in an animal model of chronic COPD. Here, we assessed the therapeutic effects of PJH in a mouse model of COPD induced by CS combined with LPS (CS/LPS) and investigated potential mechanisms. In the present work, PJH showed protective effects against inflammatory disease progression in a CS/LPS-induced COPD mouse model.

## Materials and methods

### Preparation of PJH

PJH granules were obtained from Korea Syntex Pharmaceutical (Jeonbuk, South Korea) and stored in the Herbal Medicine Research Division of the Korea Institute of Oriental Medicine (Daejeon, South Korea). For UPLC-DAD-MS/MS analyses, PJH was dissolved in methanol to a concentration of 50 mg/mL and filtered (0.2 μm pore size). Formic acid and solvents (LC-MS grade water, methanol and acetonitrile) were purchased from Thermo Fisher Scientific (Bremen, Germany).

### UPLC and MS conditions

The composition of phytochemicals in PJH was determined using a Dionex UltiMate 3000 system equipped with a Thermo Q-Exactive mass spectrometer. UPLC-DAD-MS/MS analyses were performed according to previously reported methods [29, 30]. Analyses were conducted using an electrospray ionization (ESI) source in both positive and negative ionization modes. MS spectra were acquired in full MS-ddMS^2^ mode, and all data were acquired and processed using Xcalibur v.3.0 and TraceFinder v.3.2 software (Thermo Fisher Scientific).

### Animals

Seven- week old male C57BL/6 J mice were purchased from Orient Bio (Seongnam, South Korea). Animals were acclimatized for 7 days prior to the start of dosing and were kept under environmentally constant conditions (22 °C ± 2 °C, 50% ± 5% humidity, 12-h light/dark cycle) throughout the experimental period. All animals were provided sterilized tap water and normal rodent chow *ad libitum*. Study procedures involving experimental animals were approved by the Animal Experimental Ethics Committee of Chungnam National University (Daejeon, South Korea).

### CS/LPS-induced mouse model of COPD

Animals were randomly assigned into the following five groups (n = 7–8 per group): (1) normal control (NC group); (2) CS exposure combined with LPS intranasal administration (CS + LPS group); (3) CS/LPS administration plus roflumilast 5 mg/kg oral gavage (RO; positive control group); (4) CS/LPS administration plus PJH 100 mg/kg oral gavage (PJH 100 group); and (5) CS/LPS administration plus PJH oral gavage 200 mg/kg (PJH 200 group). 3R4F research cigarettes were obtained from the Tobacco and Health Research Institute at the University of Kentucky (Lexington, KY, USA). For CS exposure, mice housed in an exposure chamber were provided fresh room air or CS from eight cigarettes for 60 min/day. The CS lasted for 8 weeks with 5 days in a week. LPS was delivered at a dose of 10 μg per mouse, administered intranasally under systemic anesthesia on weeks 1, 3, 5, and 7 (total 4 times). Roflumilast and PJH were administrated by oral gavage 1 h before CS exposure for the last 4 weeks. All animals were sacrificed by 1% pentobarbital solution (100 mg/kg).

### Collection of blood and broncho-alveolar lavage fluid (BALF)

Whole blood was collected by cardiac puncture and then centrifuged. Collected serum was frozen at −70 °C and used for further analysis. BALF was obtained by lavaging lungs three times by instilling and withdrawing 1.5 mL of phosphate-buffered saline (PBS) via a tracheal cannula. BALF supernatant was transferred to a new tube and rapidly frozen and stored at −70 °C for further analysis. Cell counts were obtained by first staining cells with trypan blue and then counting viable (i.e., unstained) cells using at least five regions of a hemocytometer under x200 and x400 magnification. BALF (100 μL) were transferred on a slide, centrifuged using a Cytospin system (Hanil Science Industrial, Gimpo, Korea), and then fixed and stained using Diff-Quik Stain reagents (B4132-1A; IMEB Inc., San Marcos, CA, USA).

### Histopathological analysis

For histopathological examinations, tissue sections were prepared as previously described [31]. Briefly, lung tissues were fixed using 10% formalin, then embedded within paraffin, and then cut into 4-μm sections. Tissue sections were stained with H&E for histological analysis. For the analysis of collagen deposition in the lung, paraffin sections were stained with Sirius red, counterstained with Mayer’s hematoxylin, and analyzed via light microscopy.

### Immunohistochemistry (IHC) assay

IHC was performed as previously described [31]. Briefly, primary antibody against matrix metallopeptidase (MMP)-7 (Abcam, Cambridge, UK) was applied overnight in 200:1 diluted solution according to manufacturer’s guide. The positive area calculated in each lung tissues of at least 5 animals per group.

### Enzyme-linked Immunosorbent assay (ELISA)

According to the manufacturer’s instructions, we performed ELISA for determination of the cytokines. The concentrations of IL-6 and TNF-α in BALF were detected using commercial ELISA kits (R&D System, MN, USA). Absorbance was measured at 450 nm using a microplate reader (Infinite m200pro; Tecan Life Sciences, Männedorf, Switzerland).

### RNA extraction and qPCR analysis of mRNA expression

Total RNA was extracted using an RNeasy mini kit (Qiagen, MD, USA). The purity of isolated RNA was evaluated based on the A_260_/A_280_ ratio, and the concentration of total RNA was evaluated based on the absorbance at 260 nm. Quantitative RT-PCR was performed using an Applied Biosystems 7500 Real-Time PCR System (Life Technologies, CA, USA) and SYBR Green PCR Master Mix (Life Technologies) as described in the manufacturer’s manual. The following PCR primer pairs were used: TNF-α, 5′-GTC TGT GCC TCA GCC TCT TC-3′ (forward) and 5′-CCC ATT TGG GAA CTT CCC T-3′ (reverse); IL-6, 5′-TAG TCC TTC CTA CCC CAA CT-3′ (forward) and 5′-TTG GTC CTT AGC CAC TCC TT-3′ (reverse); IL-1β, 5′-AGG ACC CAA GCA CCT TCT TT-3′ (forward) and 5′-AGAC AGC ACG AGG CAT TTT-3′ (reverse); TGF-β, 5′-TTG CTT CAG CTC CAC AGA GA-3′ (forward) and 5′-TGG TTGT AGA GGG CAA GGA C-3′ (reverse); MMP-7, 5′-GTT TTT GAT GCT ATT GCT GA-3′ (forward) and 5′-CCC ACA TTT GAC GTC CAG TCC AGA G-3′ (reverse); MMP-9, 5′-ACG ACA TAG ACG CCA TCC AGT-3′ (forward) and 5′-AGG TAT AGT GGG ACG ACT GGG-3′ (reverse); and GAPDH, 5′-ACA GCA ACA GGG TGG TGG AC-3′ (forward) and 5′-TTT GAG GGT GCA GCG AAC TT-3′ (reverse). Quantitative PCR results were analyzed using Applied Biosystems 7500 Real-Time PCR System software (Applied Biosystems, CA, USA) and are presented as fold-change in the cDNA level of the target gene relative to that of the endogenous constitutive control (GAPDH), determined using the 2-ΔΔC_t_ method as described previously [32].

### Western blot analysis

Equal amounts of total lung protein (35 μg) were resolved by sodium dodecyl sulfate-polyacrylamide gel electrophoresis (SDS-PAGE) and then transferred to polyvinylidene fluoride (PVDF) membranes at 35 V for 2 h. After blocking, membranes were incubated overnight at 4 °C with anti-NF-κB, anti-phospho-NF-κB, anti-ERK1/2, anti-phospho-ERK1/2 (Cell Signaling Technology, MA, USA) or anti-β-actin (Sigma Aldrich, MO, USA) antibodies. The membranes were washed with phosphate-buffered saline containing 0.1% Tween-20 (PBS-T) and then incubated with horseradish peroxidase (HRP)-conjugated secondary antibody. After washing with PBS-T, membranes were developed using an enhanced chemiluminescence (ECL) kit (Thermo Scientific, MA, USA).

### Statistical analysis

Data are expressed as means ± standard deviation (SD). Statistical comparisons were made by one-way analysis of variance (ANOVA) using SPSS 16.0 (IBM, USA), followed by Dunnett’s multiple comparisons test. A *P*-value <0.05 was considered statistically significant.

## Results

### UPLC-DAD-MS/MS analysis of PJH

**Fig. 1:**
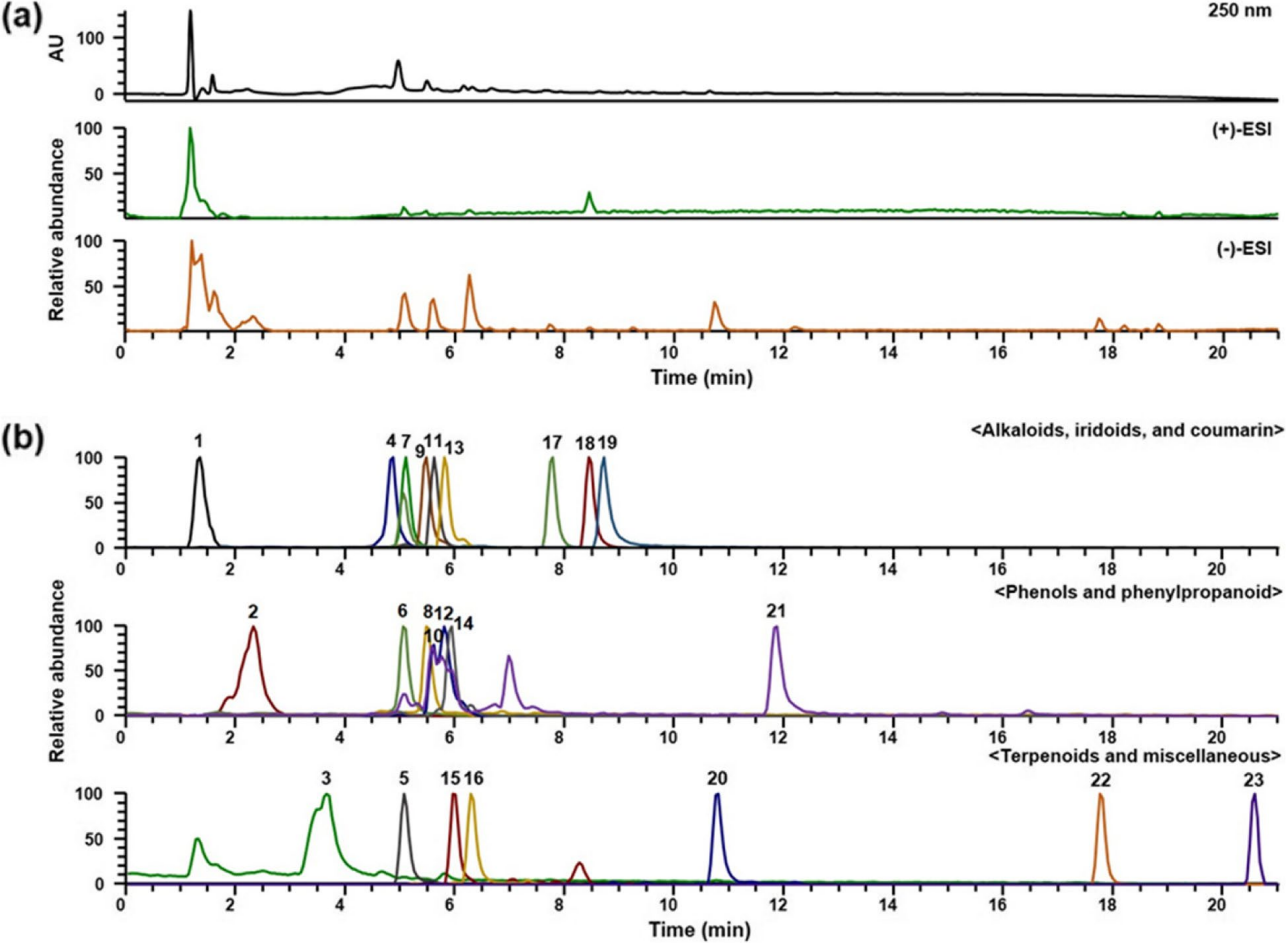
UPLC-DAD-MS/MS chromatograms of PJH. **a** LC chromatogram (250 nm) and base peak chromatograms in (+)-ESI and (−)-ESI modes. **b** Extracted ion chromatograms of the identified phytochemicals in PJH. 1, allantoin; 2, gallic acid; 3, 5-hydroxymethyl-2-furaldehyde; 4, loganic acid; 5, oxypaeoniflorin; 6, 4-hydroxybenzoic acid; 7, morroniside; 8, caffeic acid; 9, fuziline; 10, paeonolide; 11, loganin; 12, apiopaeonoside; 13, sweroside; 14, paeonoside; 15, albiflorin; 16, paeoniflorin; 17, cornuside; 18, benzoylmesaconine; 19, coumarin; 20, benzoylpaeoniflorin; 21, paeonol; 22, alisol A; 23, pachymic acid

To determine the phytochemicals in PJH, consisting of the eight herbal medicines, *Dioscorea batatas*, *Paeonia suffruticosa*, *Rehmannia glutinosa*, *Cornus officinalis*, *Aconitum carmichaelii*, *Cinnamomum cassia*, *Alisma orientale* and *Poria cocos* [33], we performed ultra-high–performance liquid chromatography coupled with diode array detection and tandem mass spectrometry (UPLC-DAD-MS/MS) analysis. The compounds in PJH were separated on an Acquity BEH C_18_ column (100 × 2.1 mm, 1.7 μm; Waters) by gradient elution using a 21-min program employing 0.1% (v/v) formic acid in water and acetonitrile. MS spectra were acquired in both (+)-ESI and (−)-ESI modes. A total of 23 compounds were identified, including allantoin from *D. batatas* [34]; gallic acid, oxypaeoniflorin, paeonolide, apiopaeonoside, paeonoside, albiflorin, paeoniflorin, benzoylpaeoniflorin and paeonol from *P. suffruticosa* [35]; 5-hydroxymethyl-2-furaldehyde and 4-hydroxybenzoic acid from *R. glutinosa* [36]; loganic acid, morroniside, caffeic acid, loganin, sweroside and cornuside from *C. officinalis* [37]; fuziline and benzoylmesaconine from *A. carmichaelii* [38]; coumarin from *C. cassia* [39]; alisol A from *A. orientale* [40]; and pachymic acid from *P. cocos* [41] (Table 1). In addition, precursor ions and MS/MS fragments of identified compounds were compared with results obtained in a previous study. Phenols, iridoids, terpenoids, and phenylpropanoid were more suitably ionized in negative ion mode, whereas other types were detected clearly in positive ion mode. The LC chromatogram at 250 nm and base peak chromatograms of PJH in positive and negative ion modes are shown in Fig. 1 together with the extracted ion chromatograms for each compound.

**Table 1:** UPLC-DAD-MS/MS analysis of phytochemicals in PJH

No.	RT (min)	Formula	Adduct	Calculated (*m*/*z*)	Measured (*m*/*z*)	Error (ppm)	MS/MS (*m*/*z*)	Identifications
1	1.34	C_4_H_6_N_4_O_3_	M-H	157.0356	157.0352	−2.9312	–	allantoin [34]
2	2.31	C_7_H_6_O_5_	M-H	169.0131	169.0128	−1.8762	169.0127, 125.0227	gallic acid [35]
3	3.66	C_6_H_6_O_3_	M + H	127.039	127.0388	−1.3138	127.0388, 109.0286	5-hydroxymethyl-2-furaldehyde [36]
4	4.81	C_16_H_24_O_10_	M-H	375.1297	375.1290	−1.7919	375.1288, 213.0756, 169.0854, 151.0745, 99.0432	loganic acid [37]
5	5.05	C_23_H_28_O_12_	M-H	495.1497	495.1497	0.0952	495.1493, 465.1373, 333.0974, 165.0540, 137.0227	oxypaeoniflorin [35]
6	5.07	C_7_H_6_O_3_	M + H	139.039	139.0385	−3.2855	139.0386, 121.0283	4-hydroxybenzoic acid [36]
7	5.09	C_17_H_26_O_11_	M + HCO_2_	451.1457	451.1447	−2.1393	243.0865, 179.0544, 155.0333, 141.0540, 123.0431	morroniside [37]
8	5.47	C_9_H_8_O_4_	M-H	179.0338	179.0337	−1.1677	179.0335, 135.0434	caffeic acid [37]
9	5.49	C_24_H_39_NO_7_	M + H	454.2799	454.2781	−3.9464	454.2783	fuziline [38]
10	5.56	C_20_H_28_O_12_	M + HCO_2_	505.1552	505.1553	0.2417	293.0879, 233.0661, 165.0541	paeonolide [35]
11	5.61	C_17_H_26_O_10_	M + HCO_2_	435.1508	435.1496	−0.2423	227.0916, 127.0378, 101.0224	loganin [37]
12	5.79	C_20_H_28_O_12_	M + HCO_2_	505.1563	505.1554	0.4229	293.0866, 165.0541	apiopaeonoside [35]
13	5.79	C_16_H_22_O_9_	M + HCO_2_	403.1235	403.1236	0.3444	357.1180, 195.0650, 179.0541, 125.0226, 81.0327	sweroside [37]
14	5.89	C_15_H_20_O_8_	M-H	327.1074	327.1079	1.3319	327.1071, 165.0541	paeonoside [35]
15	6.00	C_23_H_28_O_11_	M + H	481.1704	481.1687	−3.6772	179.0697, 151.0749, 133.0645	albiflorin [35]
16	6.26	C_23_H_28_O_11_	M + HCO_2_	525.1603	525.1600	−0.4438	449.1456, 327.1077, 165.0542, 121.0277	paeoniflorin [35]
17	7.72	C_24_H_30_O_14_	M-H	541.1563	541.1552	−1.9728	541.1540, 169.0127, 125.0226	cornuside [37]
18	8.45	C_31_H_43_NO_10_	M + H	590.2960	590.2938	0.7952	590.2941, 540.2575	benzoylmesaconine [38]
19	8.68	C_9_H_6_O_2_	M + H	147.0441	147.0436	−3.1667	147.0436, 103.0544, 91.0545, 65.0393	coumarin [39]
20	10.73	C_30_H_32_O_12_	M + HCO_2_	629.1876	629.1862	−0.4192	431.1345, 165.0542, 121.0277	benzoyl-paeoniflorin [35]
21	11.83	C_9_H_10_O_3_	M + H	167.0703	167.0698	−2.5769	167.0698	paeonol [35]
22	17.74	C_30_H_50_O_5_	M + HCO_2_	535.3629	535.3629	−0.1426	535.3597, 471.3463	alisol A [40]
23	20.56	C_33_H_52_O_5_	M-H	527.3731	527.3730	−0.2186	–	pachymic acid [41]

### Effects of PJH on CS/LPS-induced histological damage in mouse lung tissue

**Fig. 2:**
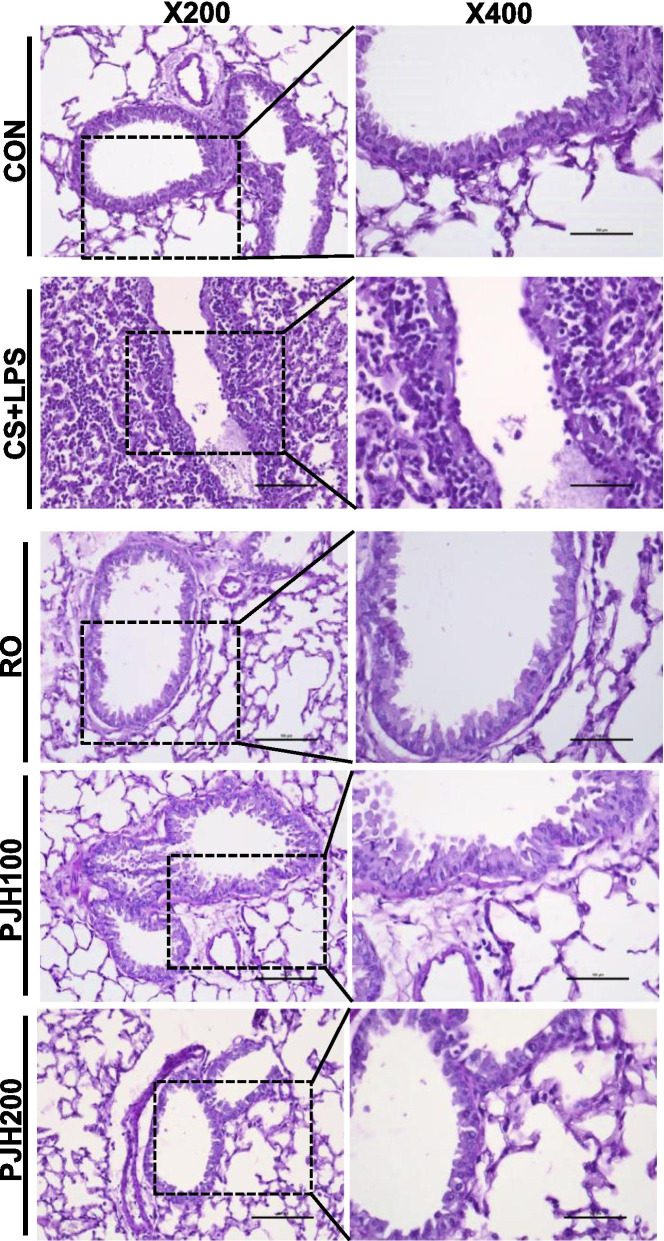
Effects of PJH on CS/LPS-induced histological changes in lung tissue. Lung tissues were fixed, sectioned at 4-μm thickness, stained with H&E, and examined under a microscope. **A** CON: control; **B** CS + LPS: CS/LPS-exposed; **C** RO: administration of roflumilast 10 mg/kg prior to CS/LPS exposure; **D** PJH 100: administration of PJH 100 mg/kg prior to CS/LPS exposure; **E** PJH 200: administration of PJH 200 mg/kg prior to CS/LPS exposure

To examine pulmonary histopathological changes resulting from treatment with PJH, we performed hematoxylin and eosin (H&E) staining. Sections of lung from the control group presented a normal bronchoalveolar phenotype with few inflammatory infiltrations (Fig. 2). Lung tissues from the CS/LPS group exhibited severe infiltration of inflammatory cells in peribronchiolar and alveolar lesions (Fig. 2). Notably, infiltration of inflammatory cells was reduced in tissues from mice treated with roflumilast or either dose of PJH compared with CS/LPS-treated mice (Fig. 2).

### Effects of PJH on the number of inflammatory cells in BALF

**Fig. 3:**
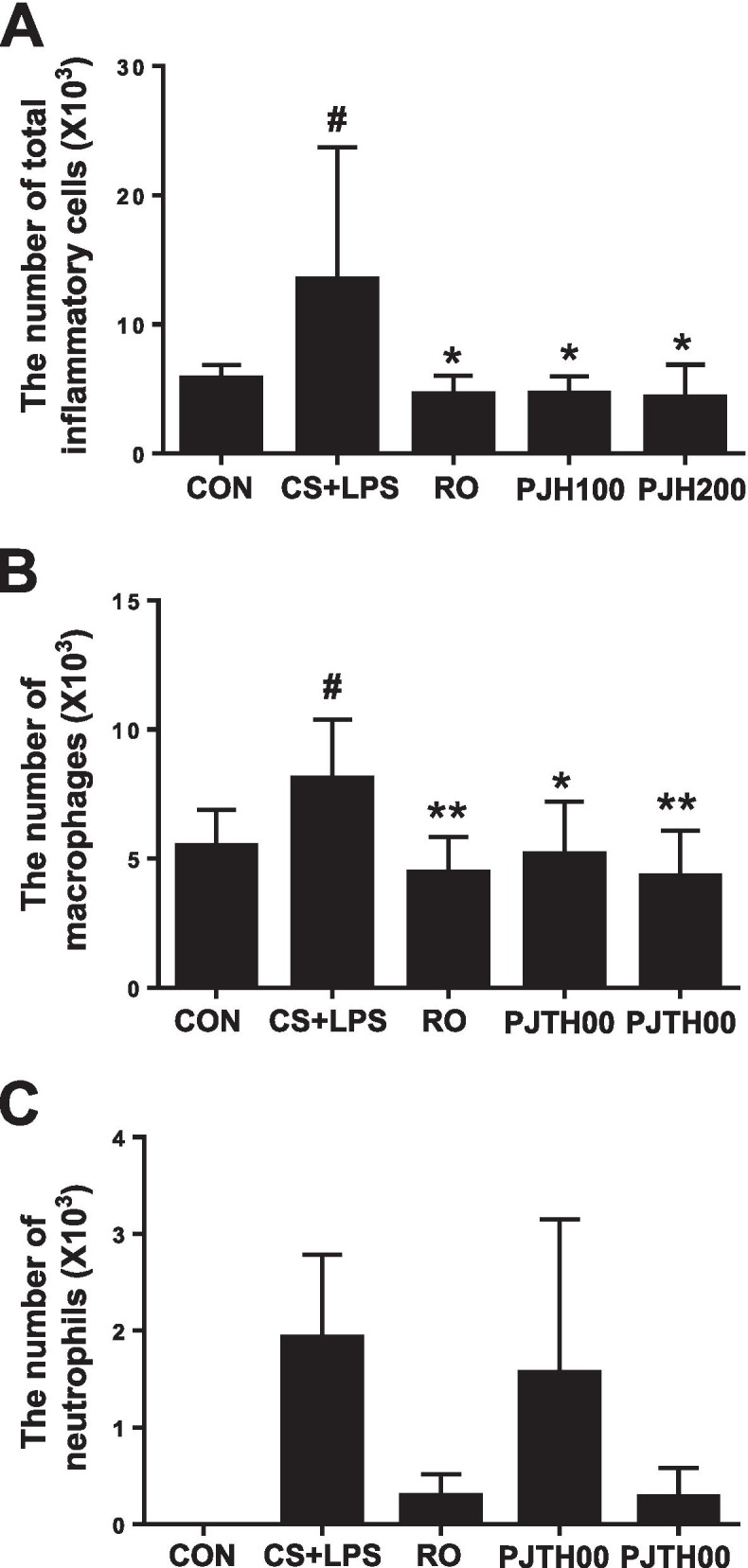
Effects of PJH on CS/LPS-induced changes in inflammatory cell counts in BALF. **A** Number of total cells in BALF. **B** Number of macrophages in BALF. **C** Number of neutrophils in BALF. CON: control group; CS + LPS group: CS/LPS-exposed; RO group: administration of roflumilast 10 mg/kg prior to CS/LPS exposure; PJH 100 group: administration of PJH 100 mg/kg prior to CS/LPS exposure; PJH 200 group: administration of PJH 200 mg/kg prior to CS/LPS exposure. Results are presented as means ± SD (^#^*P* < 0.05, ^##^*P* < 0.01 compared with the CON group; *P < 0.05, **P < 0.01 compared with the CS + LPS group)

We next evaluated effects of PJH on CS/LPS-induced pulmonary inflammation responses by performing cell counts in BALF. The number of total cells, macrophages, and neutrophils in BALF were determined. Total inflammatory cells and macrophages in BALF from the CS/LPS-treated group were significantly higher compared with those in the control group (Fig. 3A and B). In contrast, mice treated with roflumilast or either dose of PJH showed significantly decreased numbers of total cells and macrophages in BALF compared with CS/LPS-exposed animals (Fig. 3A and B). The number of neutrophil in BALF was reduced in roflumilast- and PJH-treated groups compared with CS/LPS-treated group, but the decrease was not statistically significant (Fig. 3C).

### Effects of PJH on pro-inflammatory cytokine levels in BALF

**Fig. 4:**
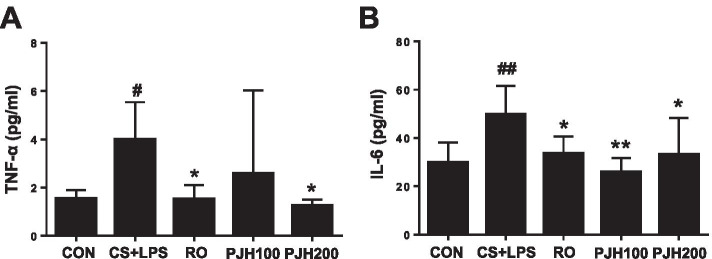
Effects of PJH on CS/LPS-induced changes in pro-inflammatory cytokines in BALF. Concentration of TNF-α (**A**) and IL-6 (**B**) in BALF. CON: control group; CS + LPS group: CS/LPS-exposed; RO group: administration of roflumilast 10 mg/kg prior to CS/LPS exposure; PJH100 group: administration of PJH 100 mg/kg prior to CS/LPS exposure; PJH200 group: administration of PJH 200 mg/kg prior to CS/LPS exposure. Results are presented as means ± SD (^#^*P* < 0.05, ^##^*P* < 0.01 compared with the CON group; *P < 0.05, **P < 0.01 compared with the CS + LPS group)

To examine the effect of PJH on pro-inflammatory cytokine production, we quantified TNF-α and IL-6 in BALF using enzyme-linked immunosorbent assays (ELISAs). Compared with the control group, CS/LPS-treated mice showed significantly elevated TNF-α and IL-6 levels in BALF (Fig. 4). Treatment with roflumilast or PJH suppressed the elevation of TNF-α and IL-6 in BALF compared with that observed in CS/LPS-exposed mice (Fig. 4).

### Effects of PJH on relative mRNA levels of cytokines in lung tissues

**Fig. 5:**
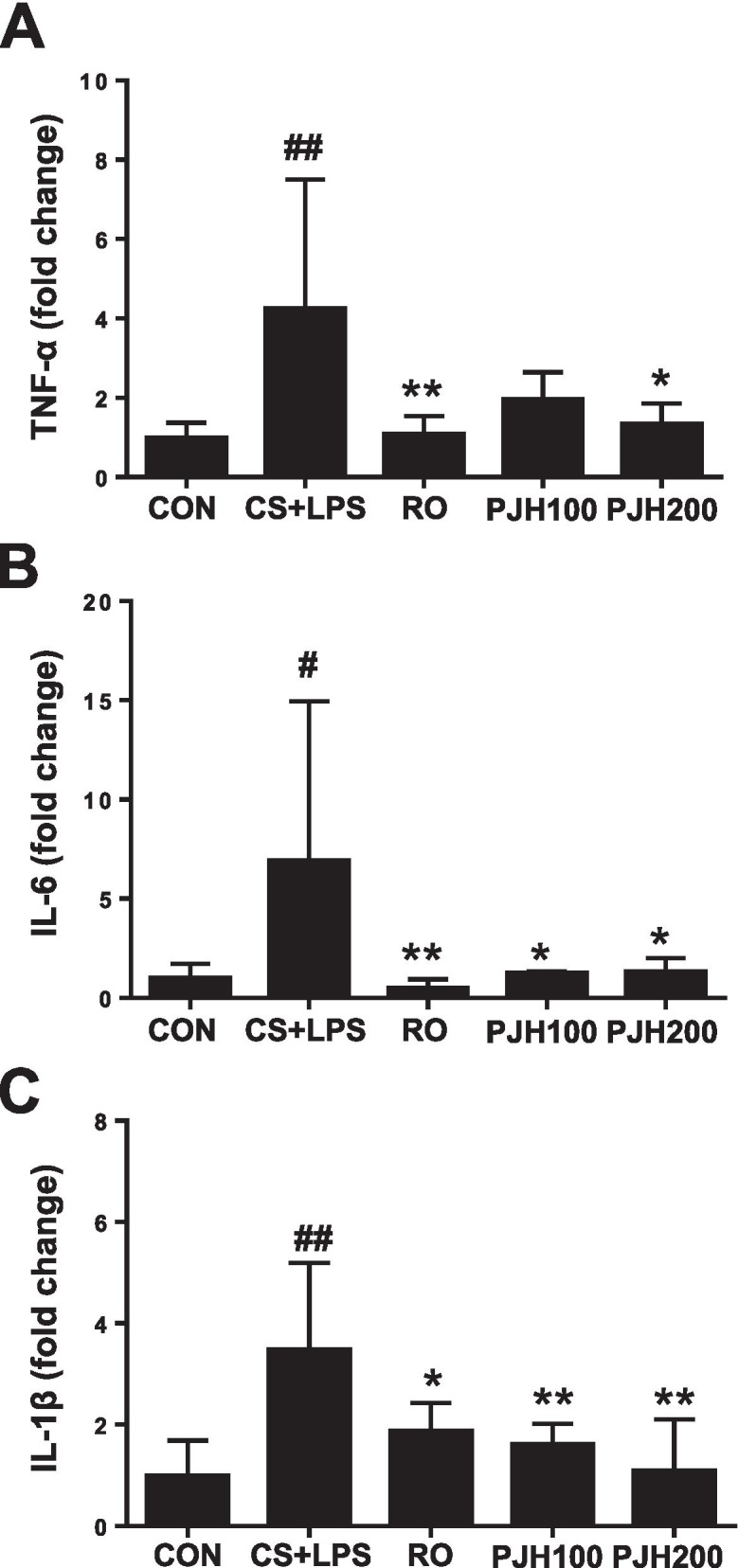
Effects of PJH on CS/LPS-induced changes in mRNA expression of cytokines in lung tissue. Quantitative PCR was performed to measure the levels of TNF-α (**A**), IL-6 (**B**) and IL-1β (**C**). CON: control group; CS + LPS group: CS/LPS-exposed; RO group: administration of roflumilast 10 mg/kg prior to CS/LPS exposure; PJH100 group: administration of PJH 100 mg/kg prior to CS/LPS exposure; PJH 200 group: administration of PJH 200 mg/kg prior to CS/LPS exposure. Results are presented as means ± SD (^#^*P* < 0.05, ^##^*P* < 0.01 compared with the CON group; *P < 0.05, **P < 0.01 compared with the CS + LPS group)

We then evaluated mRNA expression of several cytokines in lung tissue using quantitative reverse-transcription-polymerase chain reaction (qPCR). CS/LPS-exposed mice showed a notable increase in the mRNA levels of TNF-α, IL-6 and IL-1β compared with control mice (Fig. 5). Consistent with their effects in BALF, roflumilast and PJH significantly decreased IL-1β, IL-6 and TNF-α mRNA levels in lung tissues compared with those in CS/LPS-exposed mice (Fig. 5).

### Effects of PJH on NF-κB and ERK1/2 pathways

**Fig. 6:**
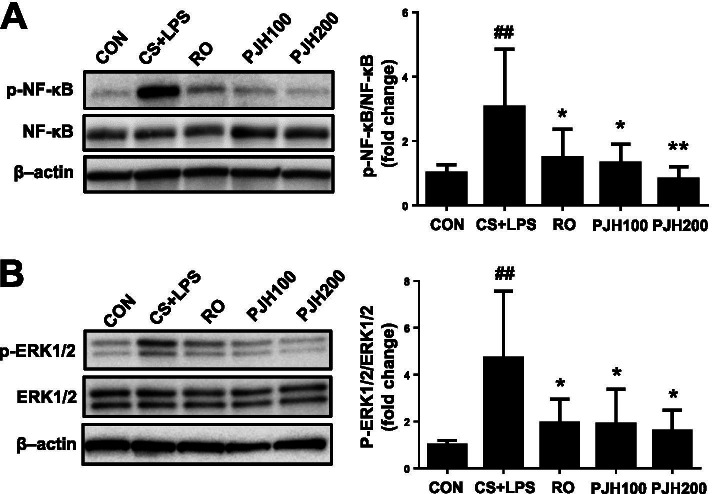
Effects of PJH on CS/LPS-induced changes in NF-κB and ERK1/2 activation. Western blot analysis was performed in lung tissues to examine the phosphorylation of NF-κB and ERK1/2. CON: control group; CS + LPS group: CS/LPS-exposed; RO group: administration of roflumilast 10 mg/kg prior to CS/LPS exposure; PJH100 group: administration of PJH 100 mg/kg prior to CS/LPS exposure; PJH200 group: administration of PJH 200 mg/kg prior to CS/LPS exposure. Results are presented as means ± SD (^#^*P* < 0.05, ^##^*P* < 0.01 compared with the CON group; *P < 0.05, **P < 0.01 compared with the CS + LPS group). Gels/blots are cropped for a clear presentation of results. Full-length blots/gels are presented in Supplementary Fig. 1. Protein samples derived from the same experiment and gels/blots were processed in parallel

Western blot analyses showed that phosphorylation of the p65 NF-κB subunit increased markedly in lung tissue from CS/LPS-treated mice. In contrast, tissues from roflumilast- or PJH-treated animals showed no increase in p65 NF-κB phosphorylation (Fig. 6A). Similarly, phosphorylated ERK1/2 was increased in CS/LPS-exposed mice, an effect that was reversed in roflumilast- and PJH-treated animals (Fig. 6B).

### PJH effects on structural changes in the lung induced by CS/LPS treatment

**Fig. 7:**
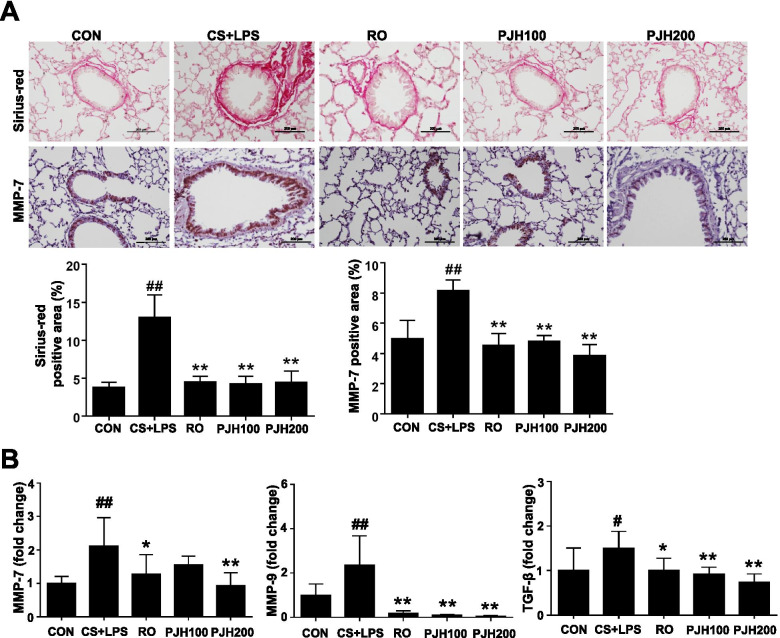
Effects of PJH on CS/LPS-induced tissue remodeling. **A** Sirius-red staining and immunohistochemistry of MMP-7 (X200). **B** Quantitative real-time PCR of MMP-7, MMP-9, and TGF-β in lung tissue. CON: control group; CS + LPS group: CS/LPS-exposed; RO group: administration of roflumilast 10 mg/kg prior to CS/LPS exposure; PJH100 group: administration of PJH 100 mg/kg prior to CS/LPS exposure; PJH200 group: administration of PJH 200 mg/kg prior to CS/LPS exposure. Results are presented as means ± SD (^#^*P* < 0.05, ^##^*P* < 0.01 compared with the CON group; *P < 0.05, **P < 0.01 compared with the CS + LPS group

Tissue remodeling in small airway walls and alveoli are key pathologies of COPD [42]. The major structural changes in tissue in COPD lungs include increased interstitial extracellular matrix (ECM) deposition and peri-bronchiolar fibrosis. Sirius-red staining showed increased peribronchiolar collagen deposition in the lungs of CS/LPS-exposed mice and this elevation were significantly alleviated in mice treated with roflumilast or PJH (Fig. 7A). In addition, the positive area of MMP-7 was increased in CS/LPS treated animals compared to control group, but this elevation was attenuated in roflumilast or PJH-treated animals (Fig. 7A). Consistently, treatment with CS/LPS increased mRNA expression of structural remodeling markers, including MMP-7 and MMP-9. In addition, the relative expression of TGF-β, a marker of lung fibrosis, was increased by CS/LPS treatment. Again, expression of these markers was repressed in roflumilast- and PJH-treated animals compared with animals in the CS/LPS-treatment group (Fig. 7B).

## Discussion

COPD is a chronic and incompletely reversible inflammatory disease characterized by progressive airflow limitations, mucous overproduction, chronic bronchitis, and remodeling of small airways [1]. Chronic exposure to CS is a major factor in the development of COPD [5]. CS not only induces extensive airway inflammation but also promotes collagen deposition in lung tissue, resulting in pulmonary fibrosis [5]. In the present study, we evaluated the protective effects of PJH in a CS/LPS-induced mouse model of COPD. Treatment with PJH markedly decreased the infiltration of inflammatory cells and production of proinflammatory cytokines in BALF and lung tissue induced by CS/LPS exposure. PJH also significantly suppressed CS/LPS-induced increases in MMP-7, MMP-9, and TGF-β levels and collagen deposition.

Inflammation is a key contributing factor to lung disease and airflow limitations in COPD [43]. Especially, CS encompass innate immune, which release pro-inflammatory cytokines and chemokines, such as TNF-α, IL-6, IL-1β and IL-8 by airway epithelial cells and alveolar macrophages. These cytokines elicit the expression of adhesion molecules on endothelial cells and the recruitment of inflammatory macrophages to the lungs [43, 44]. TNF-α, can induce the production of CXCL10, a potent chemoattractant for leukocytes such as monocytes, neutrophils and Th1 cells, and thus induces pulmonary emphysema [45, 46]. Macrophages produce IL-6, which has neutrophil chemotactic effects and is related to the severity of disease [45, 47]. IL-1β causes disruption of elastin layer fibers in alveolar septa, fibrosis in airway walls, enhanced mucin production, and formation of lymphocyte aggregates in airways [48]. In the present study, PJH treatment significantly attenuated inflammatory cell infiltration into lung tissue and reduced inflammatory cell counts (total cells and macrophages) in BALF. Furthermore, PJH reduced the production of proinflammatory cytokines, including TNF-α, IL-6 and IL-1β, in BALF and lung induced by CS/LPS exposure. These effects of PJH are consistent with those observed in a previous study by Jin *et al*., who showed that PJH inhibits the production of inflammatory mediators and cytokines in LPS-treated RAW 264.7 cells [49]. These observations, taken together with our findings, suggest that PJH suppresses the inflammatory response associated with COPD.

TNF-α, IL-6 and IL-1β appear to amplify inflammation in COPD, in part through the activation of the transcription factor, NF-kB and MAPK, thereby leading to the increased expression of multiple inflammatory genes [50]. Previous studies have demonstrated that NF-κB and MAPK are activated in bronchial biopsies and inflammatory cells of COPD individuals [51]. In addition, CS induces inflammatory cell infiltration through enhancement of NF-κB and MAPK phosphorylation [51]. Therefore, we further tested whether NF-κB and MAPK played regulatory roles in the protective effects of PJH. Consistent with these previous results, we found that CS/LPS exposure caused activation of NF-κB and ERK1/2 in lung tissue. Notably, PJH treatment evoked a significant decrease in phosphorylation of NF-κB and ERK1/2 in this mouse model of COPD, indicating that the protective effects of PJH on airway inflammatory responses induced by CS/LPS are likely closely associated with downregulation of NF-κB and ERK1/2 phosphorylation.

Small airways in COPD patients exhibit marked histological remodeling, with increased thickness of the airway wall compared with those of smokers, an increase attributable to changes in epithelial cells, increases in inflammatory cells, hyperplasia of smooth muscle tissue, and fibrosis [52]. Airway remodeling is mediated by a wound healing mechanism triggered by damage induced by CS and/or bacterial infection. The wound healing mechanism is controlled by interactions between the immune response and remodeling of extracellular matrix (ECM) by myofibroblasts in response to these stimuli [53]. The major structural changes in COPD lungs include increased interstitial ECM deposition and peri-bronchiolar fibrosis. Published experimental results suggest that the levels of several MMPs are increased in chronically inflamed lung tissues in disease states such as COPD [54]. Macrophages express numerous MMPs, and alveolar macrophages from COPD patients release large amounts of MMP-1 and MMP-9 with increased enzymatic activity compared with those of non-smokers or healthy smokers [55]. Notably, a previous report demonstrated that inhibition of MMP-9/MMP-12 considerably suppresses emphysema morphology and small airway remodeling [56]. In this study, PJH prevented the increase in MMP-7 and MMP-9 mRNA expression, suggesting the involvement of these MMPs in the protective signaling mechanism of PJH. Furthermore, alveolar macrophages generate MMPs and ROS, which disrupt the integrity of alveolar structures and lead to the production of fibrosis mediators such as TGF-β, ultimately causing airway remodeling. TGF-β1 is a key fibrosis marker that drives airway remodeling through differentiation of fibroblasts into myofibroblasts and production of ECM components such as collagen [44, 57]. In this study, PJH administration attenuated collagen accumulation in bronchioles and suppressed the elevated levels of TGF-β in mice exposed to CS/LPS. These results show that the protective effect of PJH on COPD might be associated with its protective effects on pulmonary remodeling and fibrosis.

## Conclusions

The present results demonstrate that PJH treatment significantly reduces the inflammatory response and airway remodeling induced by CS/LPS exposure, and decreases phosphorylation of NF-κB and ERK1/2. Our study provides evidence that PJH exhibits therapeutic potential to prevent COPD.

## Supplementary Information

**Additional file 1: Supplementary Fig. 1.1** The Uncropped Blot of p-NF-κB (65 kDa). The red arrow indicates the location of target bands. Blue box indicates the cropped region of Fig. 6A. **Supplementary Fig. 1.2** The Uncropped Blot of p-NF-κB (65 kDa). The red arrow indicates the location of target bands. **Supplementary Fig. 1.3** The Uncropped Blot of NF-κB (65 kDa). The red arrow indicates the location of target bands. Blue box indicates the cropped region of Fig. 6A. **Supplementary Fig. 1.4** The Uncropped Blot of NF-κB (65 kDa). The red arrow indicates the location of target bands. **Supplementary Fig. 1.5** The Uncropped Blot of β-actin (42 kDa). The red arrow indicates the location of target bands. Blue box indicates the cropped region of Fig. 6A. **Supplementary Fig. 1.6** The Uncropped Blot of β-actin (42 kDa). The red arrow indicates the location of target bands. **Supplementary Fig. 1.7** The Uncropped Blot of p-ERK1/2 (44/42 kDa). The red arrow indicates the location of target bands. Blue box indicates the cropped region of Fig. 6B. **Supplementary Fig. 1.8** The Uncropped Blot of p-ERK1/2 (44/42 kDa). The red arrow indicates the location of target bands. **Supplementary Fig. 1.9** The Uncropped Blot of ERK1/2 (44/42 kDa). The red arrow indicates the location of target bands. Blue box indicates the cropped region of Fig. 6B. **Supplementary Fig. 1.10** The Uncropped Blot of ERK1/2 (44/42 kDa). The red arrow indicates the location of target bands. **Supplementary Fig. 1.11** The Uncropped Blot of β-actin (42 kDa). The red arrow indicates the location of target bands. Blue box indicates the cropped region of Fig. 6B. **Supplementary Fig. 1.12** The Uncropped Blot of β-actin (42 kDa). The red arrow indicates the location of target bands.

## Data Availability

All the data generated and analyzed in this study are mentioned in this manuscript.

## Abbreviations

COPD: Chronic obstructive pulmonary disease
CS: Cigarette smoke
IL-1β: Interleukin-1
IL-6: Interleukin-6
MMP-7: Matrix metallopeptidase-7
MMP-9: Matrix metallopeptidase-9
NC: Negative control
NF-κB: Nuclear factor kappa B
ERK1/2: Extracellular signal-regulated kinase1/2
p.o.: Per os
RO: Roflumilast
TGF-β: Transforming growth factor β
TNF-α: Tumor necrosis factor-α
s.c.: Subcutaneous
PJH: *Palmijihwanghwan*
