# Supplementary material for: Protective effect of Palmijihwanghwan in a mouse model of cigarette smoke and lipopolysaccharide-induced chronic obstructive pulmonary disease
Source: BMC Complement Med Ther. 2021 Nov 16;21:281. doi: 10.1186/s12906-021-03453-5 (PMC8594196; doi:10.1186/s12906-021-03453-5)
Supplement: Supplementary file 1 — Additional file 1: Supplementary Fig. 1.1 The Uncropped Blot of p-NF-κB (65 kDa). The red arrow indicates the location of target bands. Blue box indicates the cropped region of Fig. 6A. Supplementary Fig. 1.2 The Uncropped Blot of p-NF-κB (65 kDa). The red arrow indicates the location of target bands. Supplementary Fig. 1.3 The Uncropped Blot of NF-κB (65 kDa). The red arrow indicates the location of target bands. Blue box indicates the cropped region of Fig. 6A. Supplementary Fig. 1.4 The Uncropped Blot of NF-κB (65 kDa). The red arrow indicates the location of target bands. Supplementary Fig. 1.5 The Uncropped Blot of β-actin (42 kDa). The red arrow indicates the location of target bands. Blue box indicates the cropped region of Fig. 6A. Supplementary Fig. 1.6 The Uncropped Blot of β-actin (42 kDa). The red arrow indicates the location of target bands. Supplementary Fig. 1.7 The Uncropped Blot of p-ERK1/2 (44/42 kDa). The red arrow indicates the location of target bands. Blue box indicates the cropped region of Fig. 6B. Supplementary Fig. 1.8 The Uncropped Blot of p-ERK1/2 (44/42 kDa). The red arrow indicates the location of target bands. Supplementary Fig. 1.9 The Uncropped Blot of ERK1/2 (44/42 kDa). The red arrow indicates the location of target bands. Blue box indicates the cropped region of Fig. 6B. Supplementary Fig. 1.10 The Uncropped Blot of ERK1/2 (44/42 kDa). The red arrow indicates the location of target bands. Supplementary Fig. 1.11 The Uncropped Blot of β-actin (42 kDa). The red arrow indicates the location of target bands. Blue box indicates the cropped region of Fig. 6B. Supplementary Fig. 1.12 The Uncropped Blot of β-actin (42 kDa). The red arrow indicates the location of target bands. [file 12906_2021_3453_MOESM1_ESM.pptx]

## Slide 1
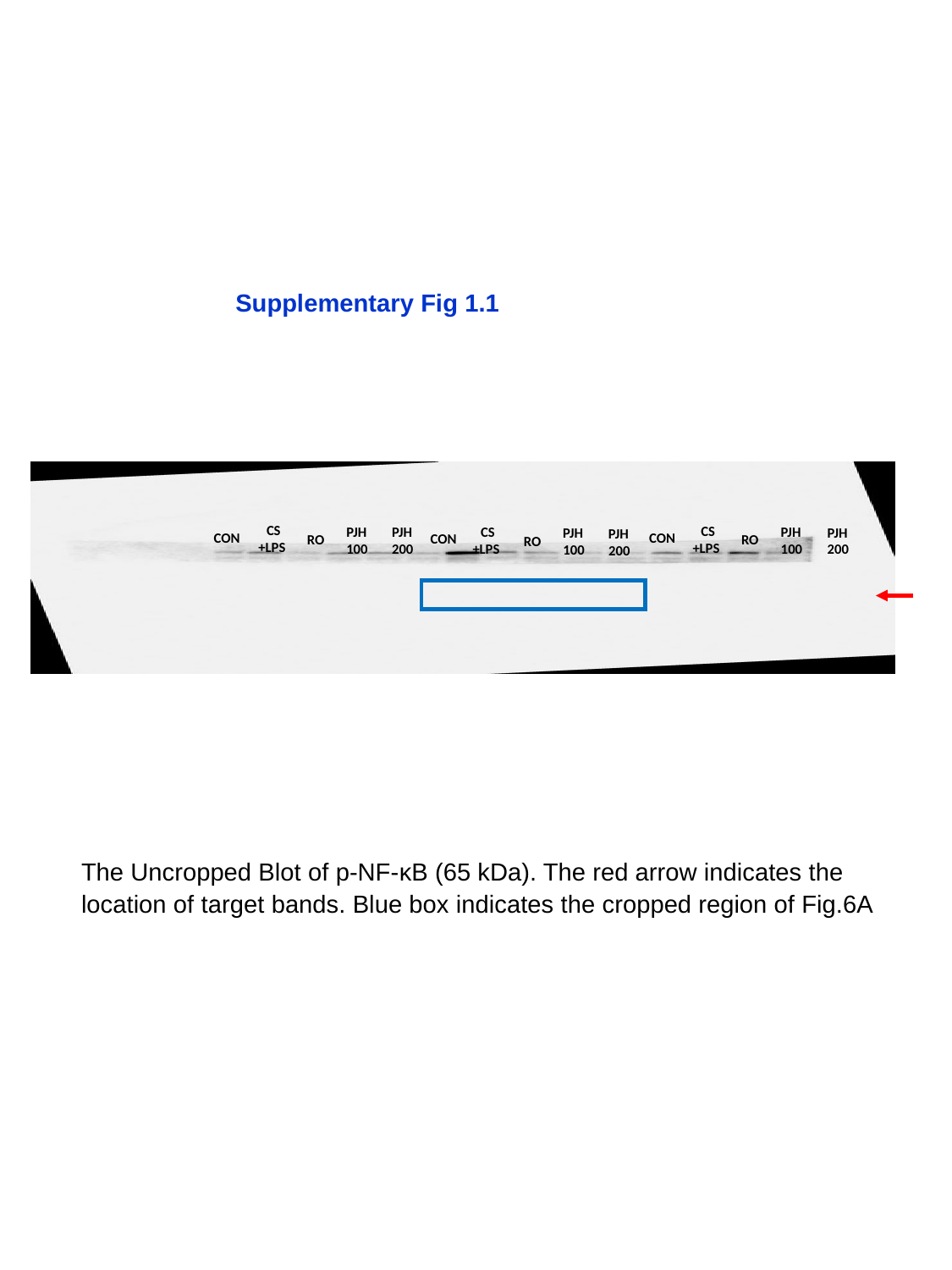

Supplementary Fig 1.1
CS
+LPS
PJH
100
PJH
200
CON
RO
CS
+LPS
PJH
100
PJH
200
CON
RO
CS
+LPS
PJH
100
PJH
200
CON
RO
The Uncropped Blot of p-NF-κB (65 kDa). The red arrow indicates the
location of target bands. Blue box indicates the cropped region of Fig.6A

## Slide 2
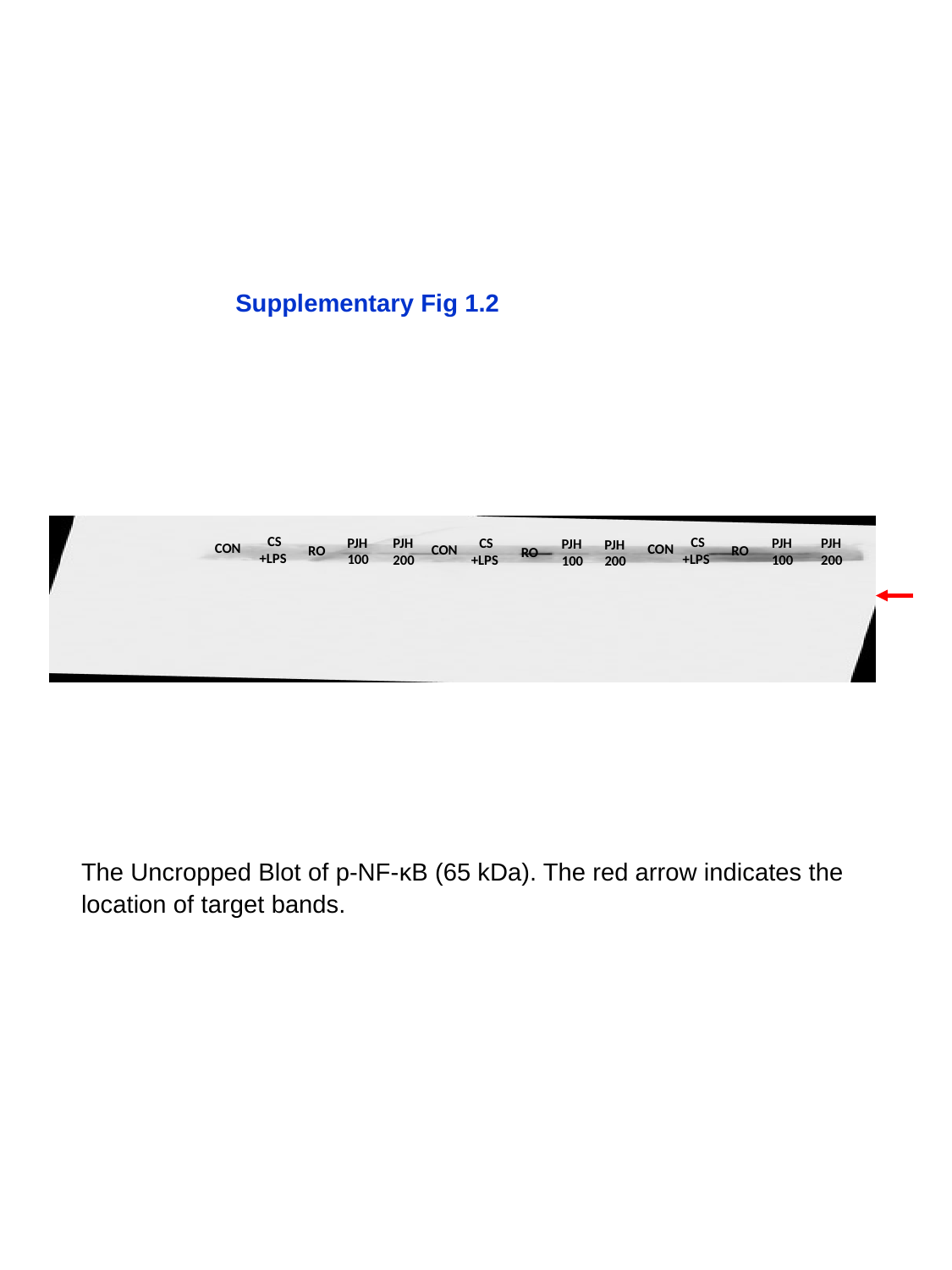

Supplementary Fig 1.2
CS
+LPS
PJH
100
PJH
200
CON
RO
CS
+LPS
PJH
100
PJH
200
CON
RO
CS
+LPS
PJH
100
PJH
200
CON
RO
The Uncropped Blot of p-NF-κB (65 kDa). The red arrow indicates the
location of target bands.

## Slide 3
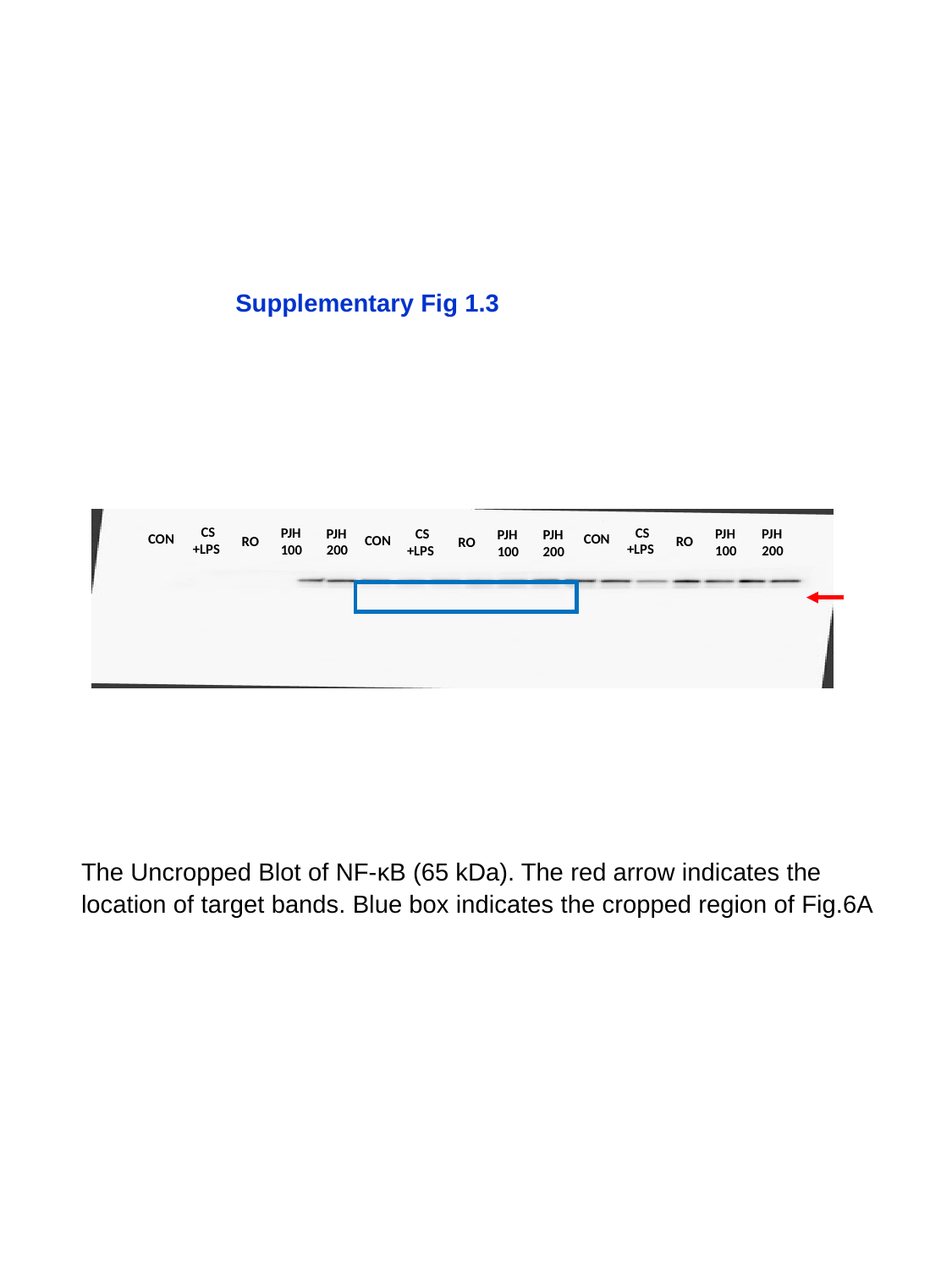

Supplementary Fig 1.3
CS
+LPS
PJH
100
PJH
200
CON
RO
CS
+LPS
PJH
100
PJH
200
CON
RO
CS
+LPS
PJH
100
PJH
200
CON
RO
The Uncropped Blot of NF-κB (65 kDa). The red arrow indicates the
location of target bands. Blue box indicates the cropped region of Fig.6A

## Slide 4
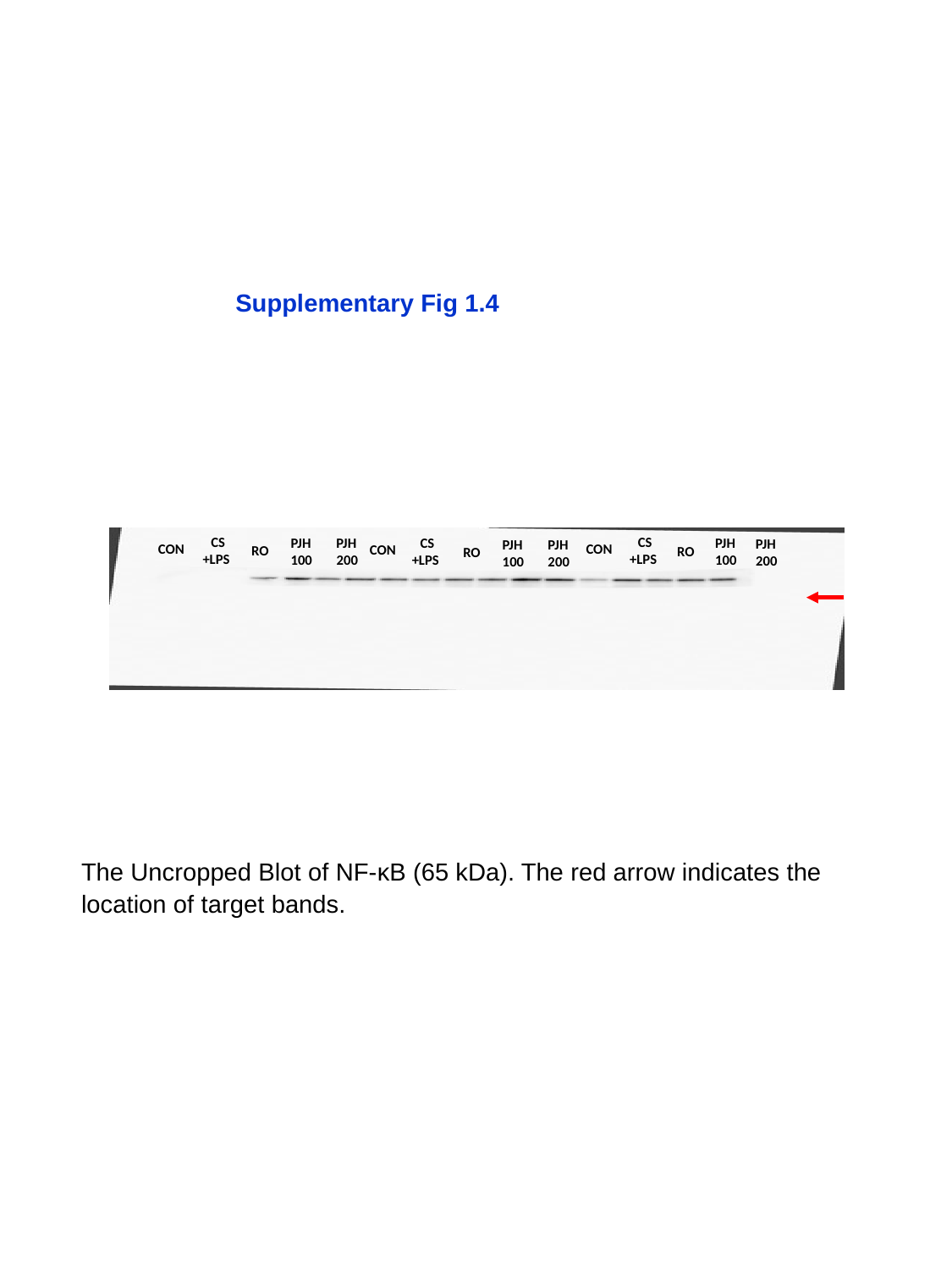

Supplementary Fig 1.4
CS
+LPS
PJH
100
PJH
200
CON
RO
CS
+LPS
PJH
100
PJH
200
CON
RO
CS
+LPS
PJH
100
PJH
200
CON
RO
The Uncropped Blot of NF-κB (65 kDa). The red arrow indicates the
location of target bands.

## Slide 5
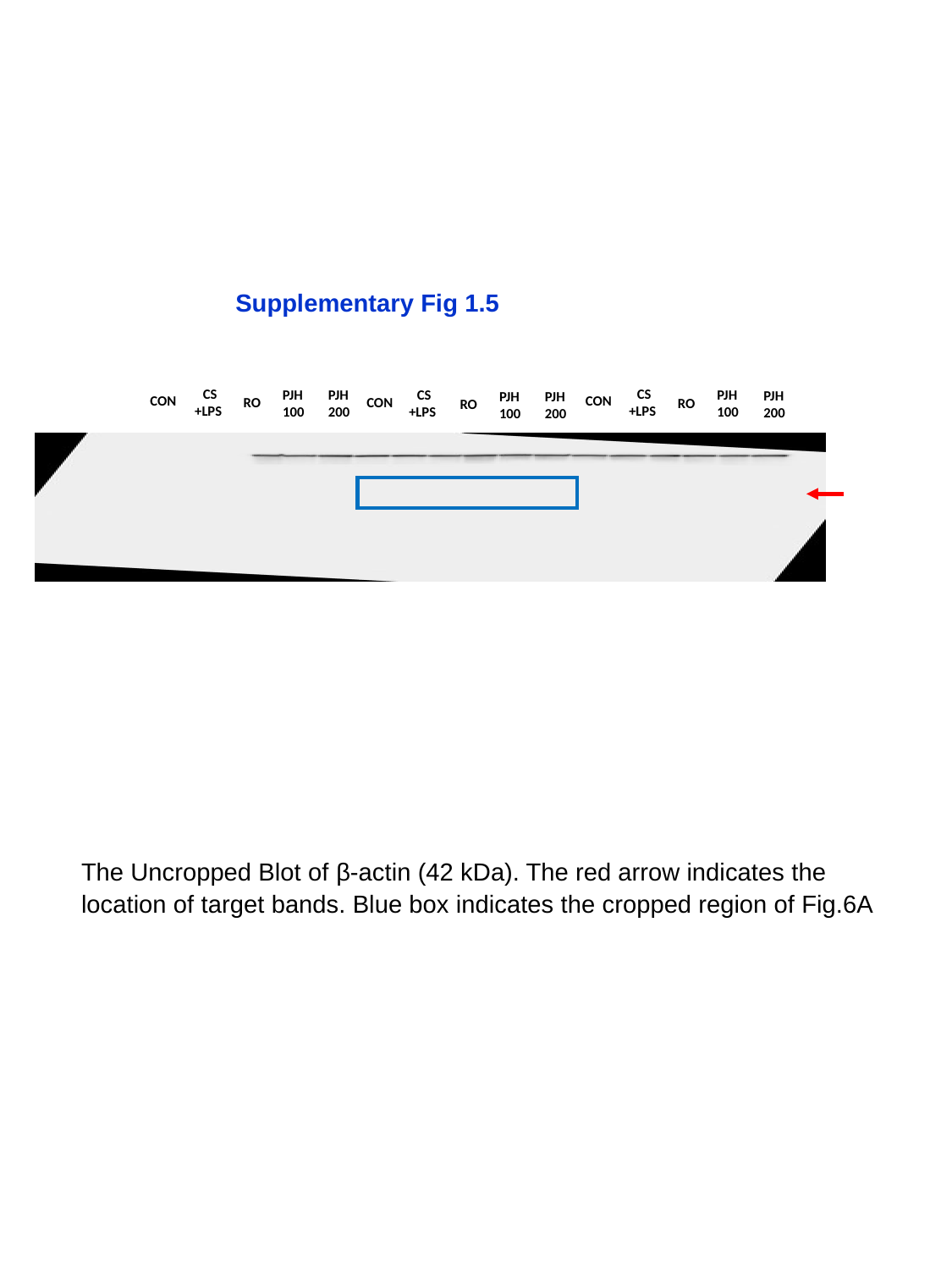

Supplementary Fig 1.5
CS
+LPS
PJH
100
PJH
200
CON
RO
CS
+LPS
PJH
100
PJH
200
CON
RO
CS
+LPS
PJH
100
PJH
200
CON
RO
The Uncropped Blot of β-actin (42 kDa). The red arrow indicates the
location of target bands. Blue box indicates the cropped region of Fig.6A

## Slide 6
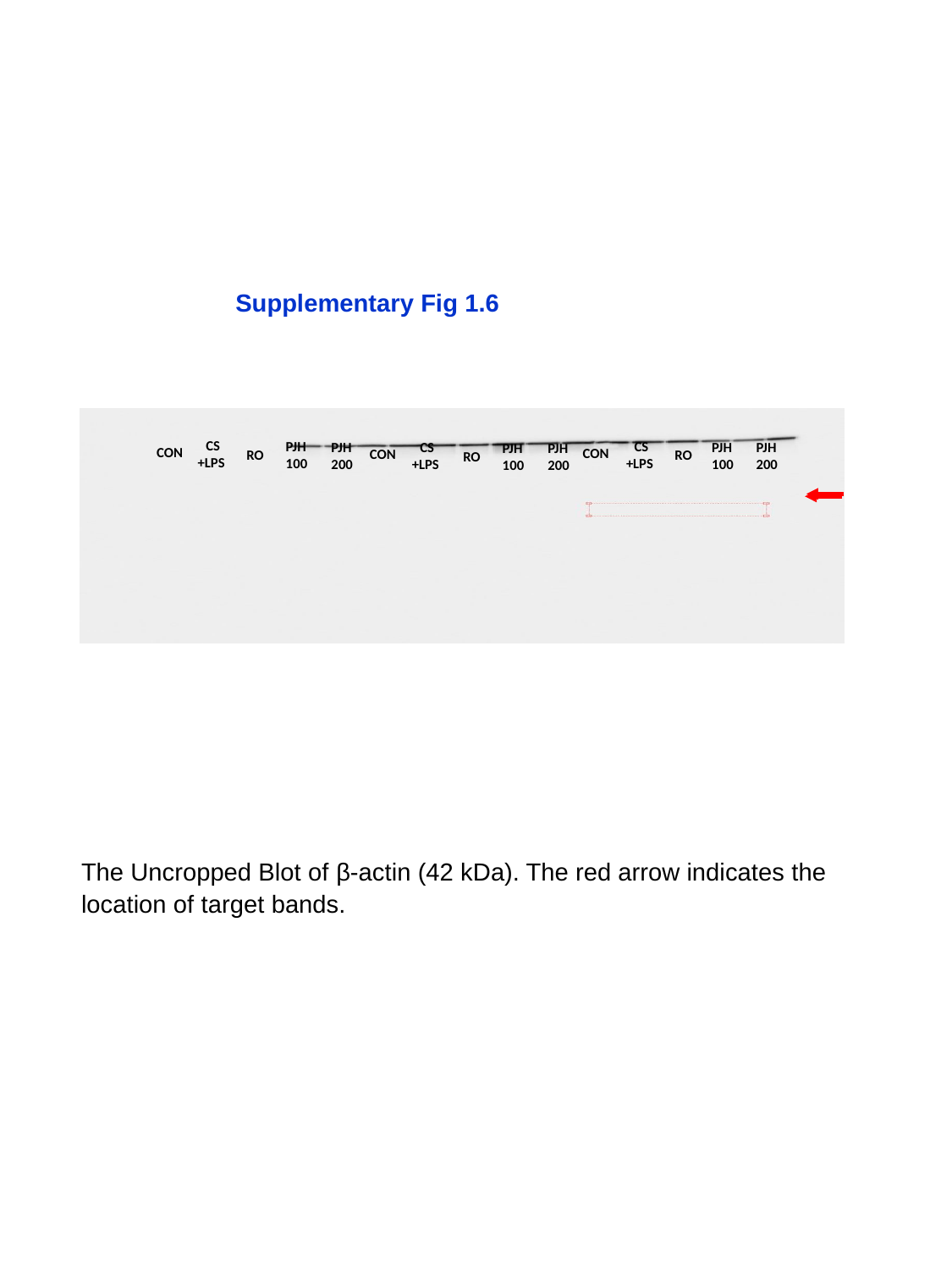

Supplementary Fig 1.6
CS
+LPS
PJH
100
PJH
200
CON
RO
CS
+LPS
PJH
100
PJH
200
CON
RO
CS
+LPS
PJH
100
PJH
200
CON
RO
The Uncropped Blot of β-actin (42 kDa). The red arrow indicates the
location of target bands.

## Slide 7
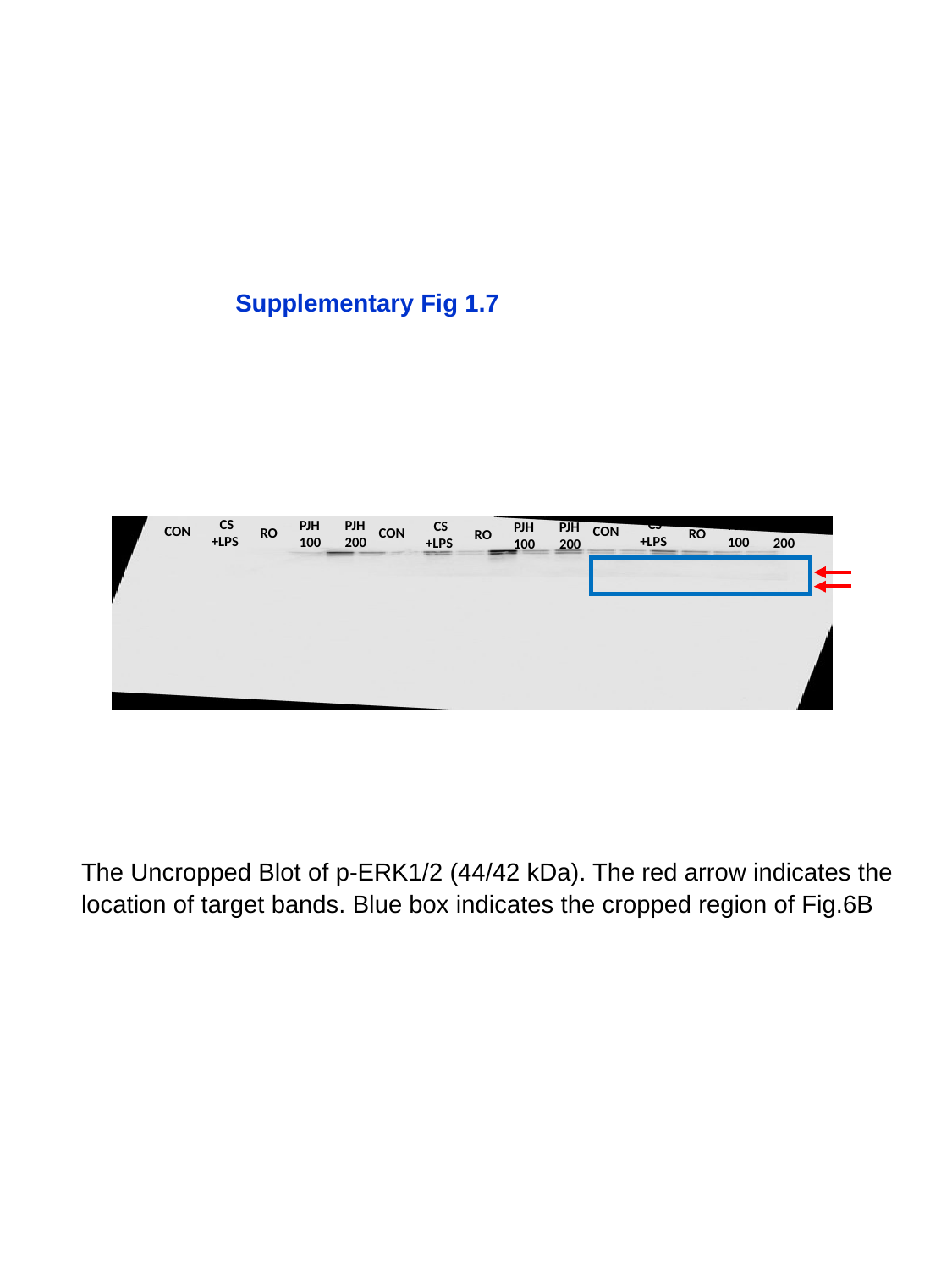

Supplementary Fig 1.7
CS
+LPS
PJH
100
PJH
200
CON
RO
CS
+LPS
PJH
100
PJH
200
CON
RO
CS
+LPS
PJH
100
PJH
200
CON
RO
The Uncropped Blot of p-ERK1/2 (44/42 kDa). The red arrow indicates the
location of target bands. Blue box indicates the cropped region of Fig.6B

## Slide 8
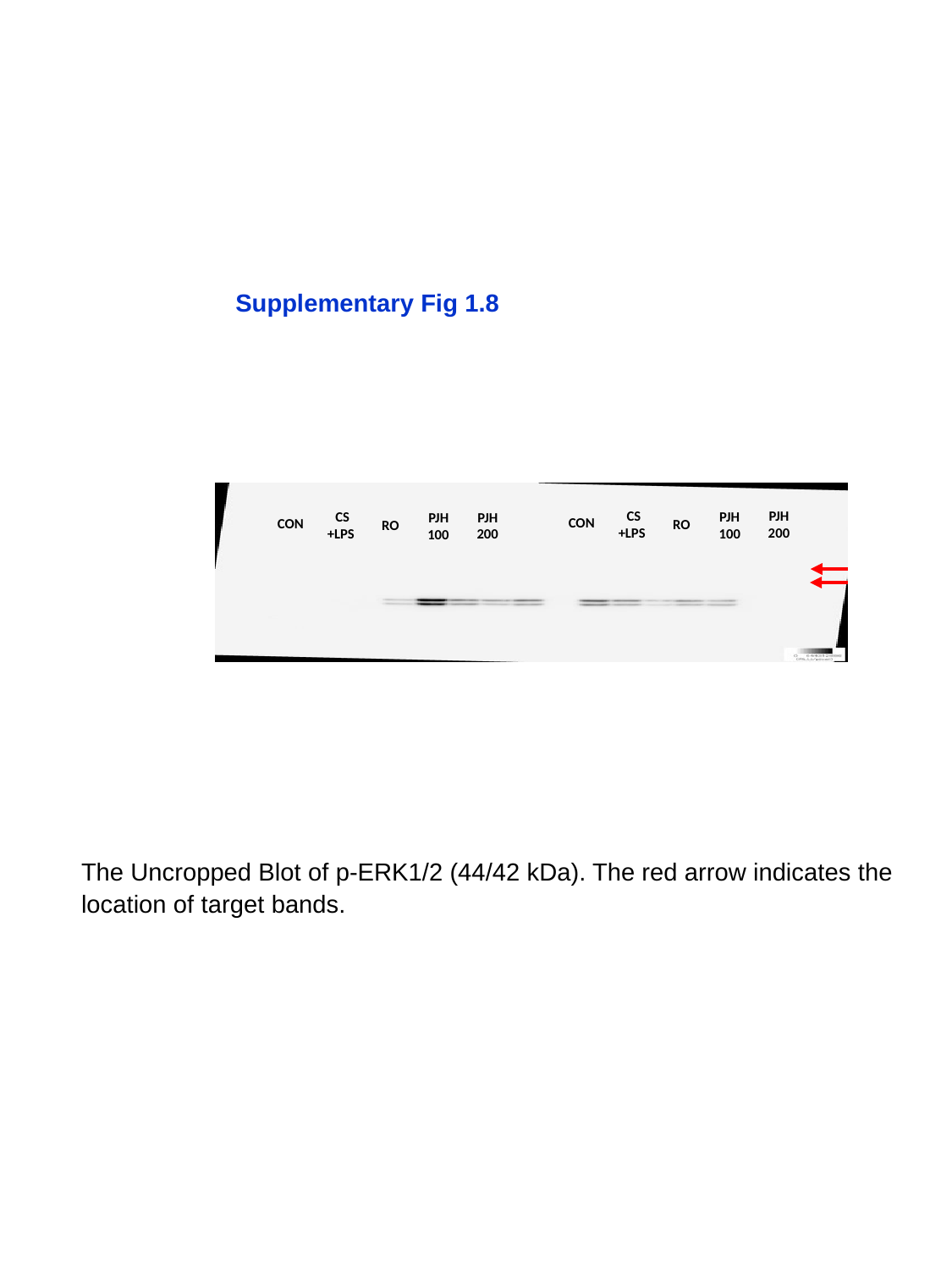

Supplementary Fig 1.8
CS
+LPS
PJH
200
PJH
100
CON
RO
CS
+LPS
PJH
200
PJH
100
CON
RO
The Uncropped Blot of p-ERK1/2 (44/42 kDa). The red arrow indicates the
location of target bands.

## Slide 9
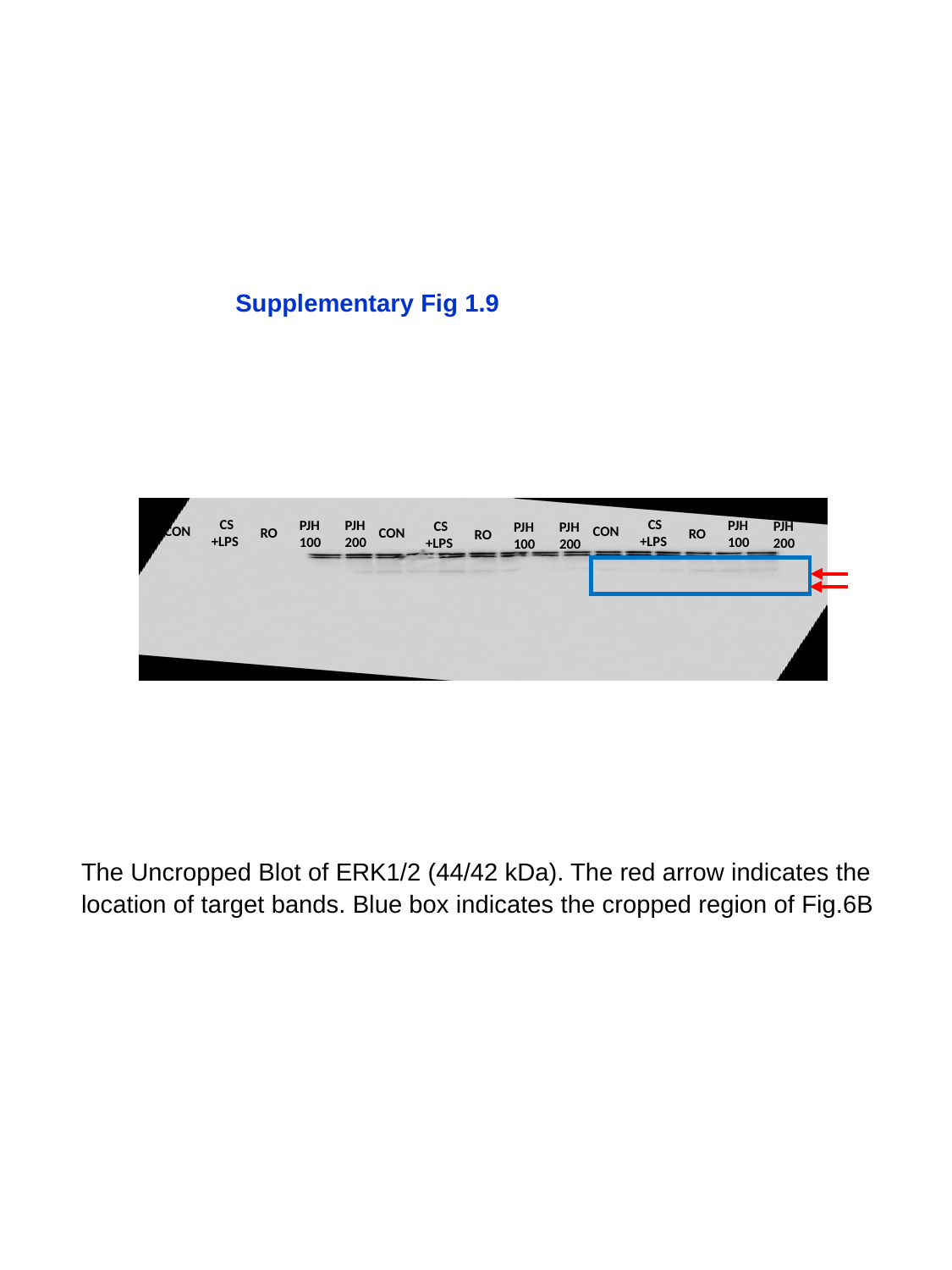

Supplementary Fig 1.9
CS
+LPS
PJH
100
PJH
200
CON
RO
CS
+LPS
PJH
100
PJH
200
CON
RO
CS
+LPS
PJH
100
PJH
200
CON
RO
The Uncropped Blot of ERK1/2 (44/42 kDa). The red arrow indicates the
location of target bands. Blue box indicates the cropped region of Fig.6B

## Slide 10
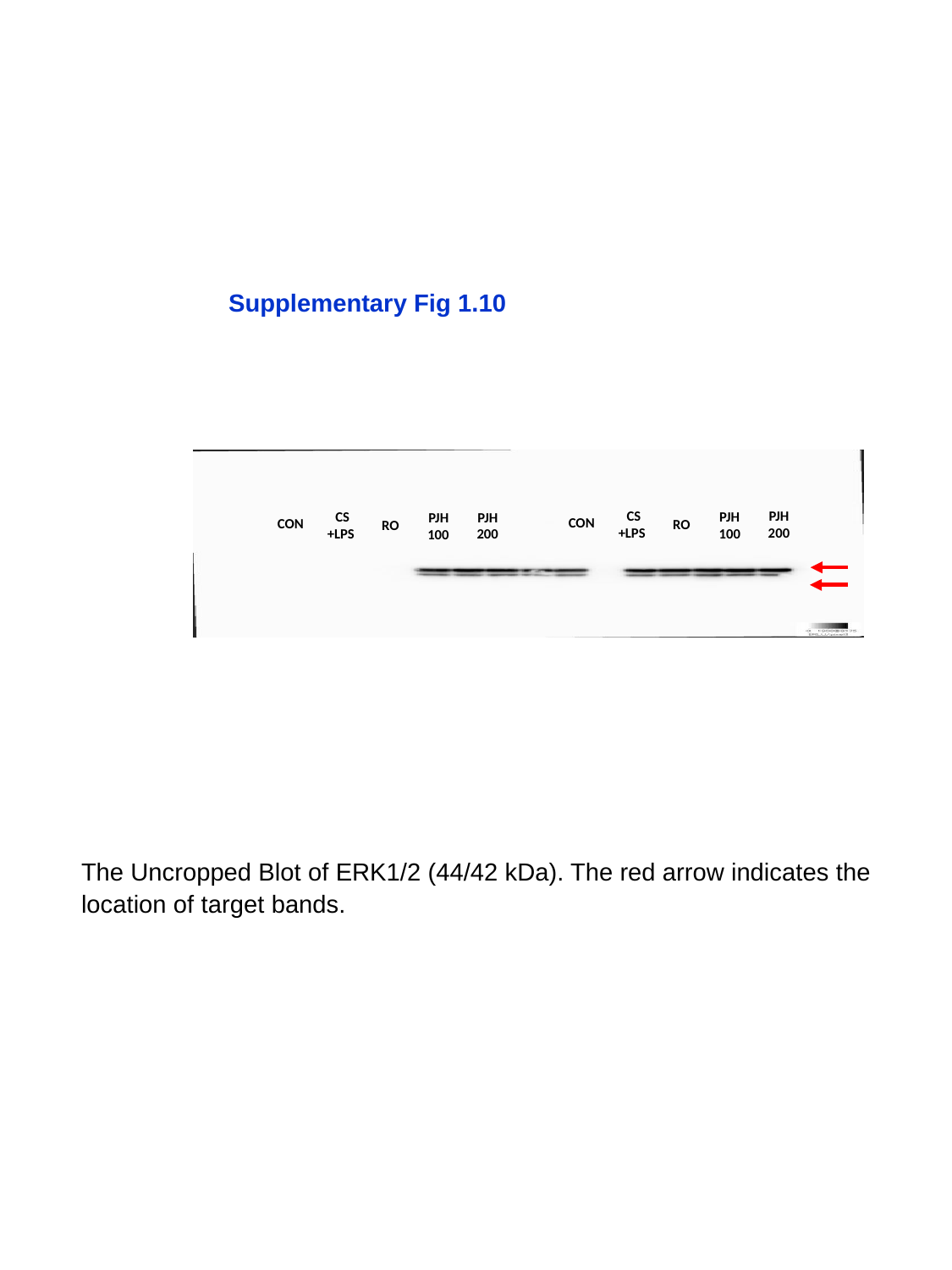

Supplementary Fig 1.10
CS
+LPS
PJH
200
PJH
100
CON
RO
CS
+LPS
PJH
200
PJH
100
CON
RO
The Uncropped Blot of ERK1/2 (44/42 kDa). The red arrow indicates the
location of target bands.

## Slide 11
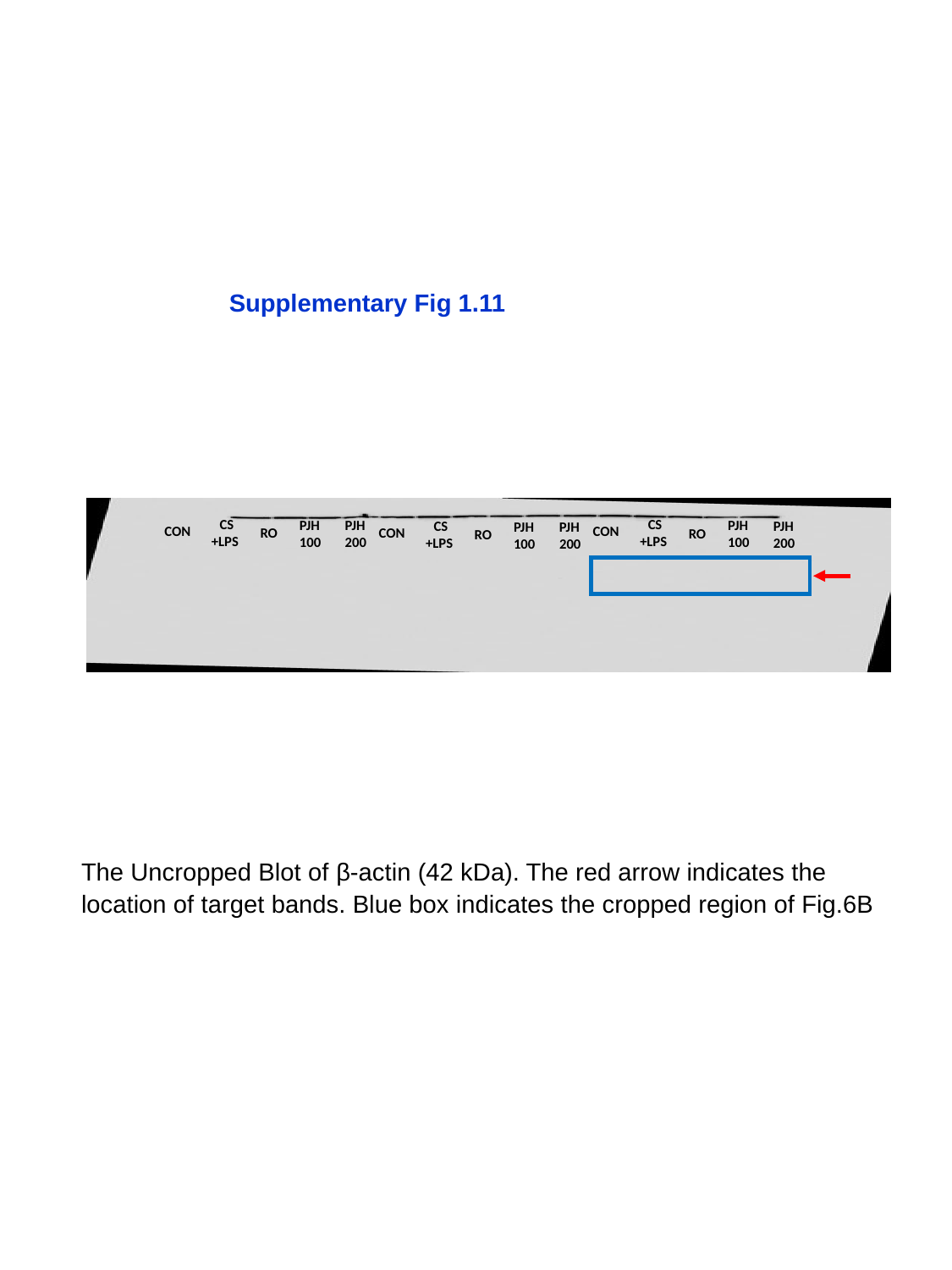

Supplementary Fig 1.11
CS
+LPS
PJH
100
PJH
200
CON
RO
CS
+LPS
PJH
100
PJH
200
CON
RO
CS
+LPS
PJH
100
PJH
200
CON
RO
The Uncropped Blot of β-actin (42 kDa). The red arrow indicates the
location of target bands. Blue box indicates the cropped region of Fig.6B

## Slide 12
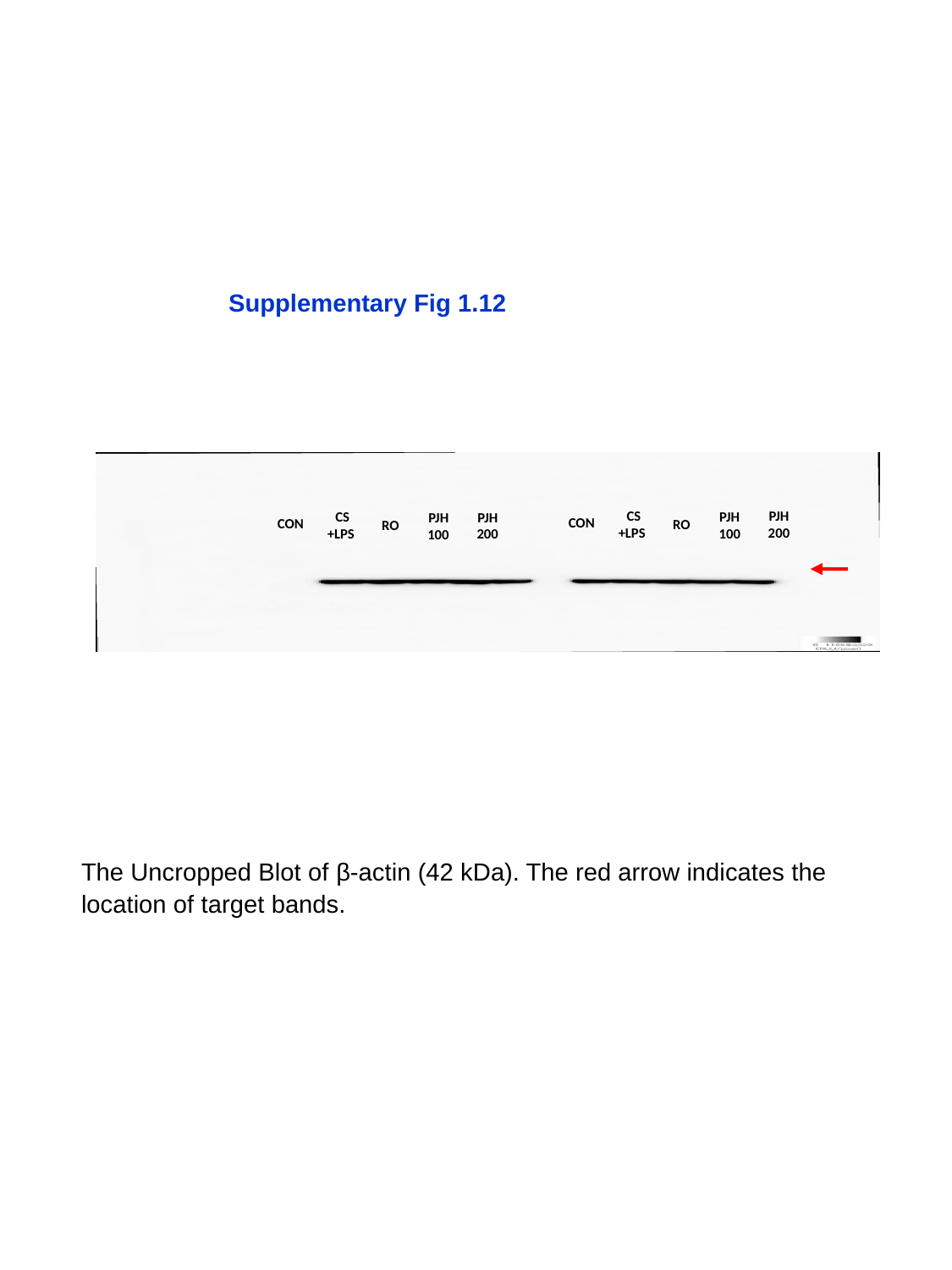

Supplementary Fig 1.12
CS
+LPS
PJH
200
PJH
100
CON
RO
CS
+LPS
PJH
200
PJH
100
CON
RO
The Uncropped Blot of β-actin (42 kDa). The red arrow indicates the
location of target bands.
